# 521 Silk A Biological Dressing for Superficial Burns Treatment: Randomized Controlled Trial Versus Silver Sulfadiazine

**DOI:** 10.1093/jbcr/irad045.118

**Published:** 2023-05-15

**Authors:** Eric J Dantzer

**Affiliations:** Burn Service Instruction Military Hospital Sainte Anne Toulon FRANCE, La Garde, Provence-Alpes-Cote d'Azur

## Abstract

**Introduction:**

Silver Sulfadiazine is currently the standard treatment for superficial second-degree burns. These burns are common and should not necessarily require hospital treatment. However, caretaking may become an issue: dressing technique is not always mastered by home health care nurses, their practical realization may be more difficult considering the surface to be treated; duration of care can also be too long and painful. We have evaluated the interest of a dressing based on natural silk with this indication.

**Methods:**

In a preliminary study, we have compared SSD to silk dressing (S) in the treatment of 2nd degree burns. Following diagnosis, the topical to be used was determined by drawing of lots. The SSD associated with fatty gauze was applied and then covered with dry gauzes and bandages. Silk was applied directly on the lesion and then covered with dry gauzes and bandages. Dressings were changed every 48 hours. The SSD was changed with each dressing after cleansing the lesion with saline; the silk one was left in place until healing, only the dry gauzes and bandages were changed. Pain, duration of care and healing time were assessed.

**Results:**

80 patients were included 25 men, 15 women (7 to 83years) were treated with SSD; 22 men, 18 women (17 to 84 years) with silk. The average surface area treated was 5% in both groups (1% to 20% SSD) and (1% to 23% S). Pain (EVA) was evaluated between 3 and 4/10 for the SSD group and 0 to 2 for the S group. Dressing duration was 20-30 minutes for the SSD group and 10-15 minutes for the S group. The average healing time was 13 days for both groups. 2 complications, as infections , were observed in each group.

**Conclusions:**

The flexibility of silk facilitates smooth application on all surfaces and localisations like SSD. Silk treatment has the same healing time as that obtained with SSD, i.e. the physiological healing time of 2^nd^ degree superficial burns. Silk has allowed shorter treatments, because it is technically simple, and less painful as the lesion was never exposed to air. This allowed reduction of analgesics. A larger study should confirm that 2nd degree burns can be managed with silk dressing as outpatients, by home health care nurses, inexperienced in burns and even for larger surfaces and reduce the cost effective of the treatment and the need for painkillers.

**Applicability of Research to Practice:**

immediate

